# Genomic analysis of *Botrytis cinerea* causing postharvest strawberry rot and the control effect of pydiflumetofen

**DOI:** 10.3389/fmicb.2026.1839241

**Published:** 2026-06-10

**Authors:** Haohao Yan, Lili Jiang, Tianyu Guo, Chao Wang, Mikael Motelica-Heino, Chong Wu

**Affiliations:** 1 Shandong Institute of Pomology, Tai’an, China; 2 College of Horticultural Science and Engineering, Shandong Agricultural University, Tai’an, Shandong, China; 3 Shandong Xinhua New Gelin Intelligent Agriculture Co., Ltd., Zaozhuang, Shandong, China; 4 Institute of Earth Sciences of Orleans, University of Orléans, Orléans, France

**Keywords:** *Botrytis cinerea*, genomic analysis, high-throughput sequencing, postharvest strawberry rot, pydiflumetofen

## Abstract

China exhibits the largest global cultivation area of delicious, highly nutritious small fruit, including strawberries. However, fruit storage is challenged by pathogen infection. In this study in Tai’an (117.09°E, 36.19°N), Shandong Province, China, we identified the pathogenic factors of *Botrytis cinerea* HM-Y1, which causes postharvest strawberry rot. We further used high-throughput sequencing to identify the main pathogens causing postharvest strawberry rot at the genus level. HM-Y1 was successfully isolated and identified as *Botrytis cinerea*. Spore morphology was assessed, and a multigene phylogenetic tree was constructed, including *ITS*, *HSP60*, *RPB2*, and *G3PDH*. To elucidate the genetic signatures of *Botrytis cinerea*, we analyzed the HM-Y1 genome. The total sequence length of *B. cinerea* HM-Y1 was found to be 43,237,401 bp, with a GC content of 41.95%. Furthermore, we found that HM-Y1 biosynthesize the terpenoid virulence factor botrydial inferred from genomic data, and that pydiflumetofen has a significant antifungal effect against this fungus, with an EC_50_ value of 0.200 mg/L. The control effect of 2 mg/L pydiflumetofen on the strawberry rot disease caused by *B. cinerea* HM-Y1 was 66.67%. These results suggest that pydiflumetofen provides effective control for the management of postharvest fungal diseases in strawberry.

## 1 Introduction

Strawberry plants (*Fragaria* × *ananassa* Duch.) are perennial herbaceous members of the Rosaceae family and produce high-value fruit rich in polyphenolic compounds, such as flavonols, anthocyanins, vitamins, fibers, and antioxidants (Duan et al., 2025; Lin et al., 2025; Liu et al., 2023; Wang L. et al., 2025; Wang Z. Y. et al., 2025). Strawberries exhibit protective effects on the cardiovascular and cerebrovascular systems, as well as antioxidation, anti-inflammation, anticancer, and antimicrobial properties (Bezerra et al., 2024; Hernández-Martínez et al., 2023). These fruits are often referred to as the “homology of medicine and food” and the “queen of berries.” Thus, they are of considerable horticultural, commercial, and nutritional importance and are highly valued by consumers (Gaston et al., 2020; Mukherjee and Gantait, 2024). The global output of strawberries has been increasing annually, reaching 9.6 million tons in 2022 (Kelanne et al., 2025). China is the biggest producer of strawberries and exhibits the largest strawberry cultivation area worldwide (Jiang et al., 2025), accounting for 5 billion USD, over 3 times the value of the second-largest producer, the United States of America (Hernández-Martínez et al., 2023).

Postharvest pathogens can grow in large numbers on strawberries, and strawberry losses can reach 50%, causing significant economic losses to farmers and retailers (Momtaz et al., 2025; Yuan et al., 2025). Strawberry fruits are prone to various diseases at different developmental stages, including during postharvest storage and transportation, which can result in irreversible losses in strawberry quality (Wang L. et al., 2025; Wang Z. Y. et al., 2025). Thus, research on postharvest preservation and strawberry disease resistance has become a prominent focus in postharvest fruit biology (Xing et al., 2025). Various postharvest treatment methods are employed to prevent microbial contamination and extend the shelf life of strawberries (Priyadarshi et al., 2024). Plant pathogenic fungi are generally highly adapted organisms and often show high degrees of host specificity. Analyzing the fungal genome enables the identification of cell death-inducing proteins (CDIPs) and phytotoxic metabolites (Klug et al., 2024).

Current postharvest disease management in strawberries primarily relies on chemical control (Yang et al., 2024; Zhao et al., 2026). Pydiflumetofen, a broad-spectrum succinate dehydrogenase inhibitor (SDHI) fungicide, developed by Syngenta, can inhibit the growth of many plant pathogens, such as *Alternaria* spp., *Blumeria* spp., *Sclerotinia* spp., and *Fusarium* spp. by interrupting fungal respiration (Li et al., 2022; Zhao et al., 2022; Zhou et al., 2024). However, the application and mechanism of pydiflumetofen in postharvest diseases remain largely unexplored (He et al., 2020).

The objectives of this study were (1) to identify pathogenic factors at the genus level through high-throughput sequencing; (2) to identify pathogenic factors causing postharvest strawberry rot inferred from genomic data; and (3) to calculate EC_50_ value and assess the control efficacy of pydiflumetofen on pathogenic fungi. This study was conducted to help growers effectively manage strawberry fruit rot, evaluate the application of pydiflumetofen for controlling postharvest diseases.

## 2 Materials and methods

### 2.1 Fungal and bacterial communities of healthy and diseased strawberry fruits

In June 2025, a localized occurrence of fruit rot was observed on harvested strawberry fruits (cv. Hongyan), with an incidence of 30% among 50 kg of examined fruits in Tai’an (117.09°E, 36.19°N), Shandong Province, China. Strawberry fruits exhibiting typical rot characteristics (Group I) and healthy characteristics (Group H) were collected. High-throughput sequencing technology was used to further determine the primary pathogen associated with strawberry rot. After thorough grinding, tissue samples (*n* = 5) were sent to Ouyi Biotechnology Co., Ltd. for ITS, 28S, and 16S high-throughput sequencing (Wang L. et al., 2025; Wang Z. Y. et al., 2025; Xu et al., 2024).

### 2.2 Identification of pathogen strain in diseased strawberry fruits

Small (1–2 mm) segments of infected tissue were obtained from five randomly selected fruits. The samples were surface-sterilized with 75% ethanol for 30 s, then with 5% sodium hypochlorite (NaClO) for 3 min, rinsed three times with sterile distilled water, and dried on paper towels. Cultures were purified using single-spore isolation and plated in 9-cm Petri dishes containing potato dextrose agar (PDA) for fungal pathogen isolation (Yan et al., 2025b; Yousef et al., 2024). A spore suspension of the fungus was prepared by washing the culture with sterile distilled water, five fruits were inoculated with 10-μL conidial suspension (10^6^ spores/mL, determined by hemocytometer) of the isolate, while five fruits treated with sterile distilled water served as controls (Yuan et al., 2024). The fruits were incubated at 25 °C and 75% relative humidity and the experiment was repeated twice. The purified strains were then maintained as glycerol suspensions (20%, v/v) at −80 °C for long-term storage. The homogenate obtained after soaking the collected fruits was streaked onto Luria-Bertani (LB) agar plates for isolation of bacterial pathogens; however, no pathogenic bacteria were isolated.

Genomic DNA was extracted from the isolated fungus with the highest pathogenicity using the Ezup Column Fungi Genomic DNA Purification kit (Sangon Biotech, Shanghai, China) for molecular confirmation (Yan et al., 2024, 2025a). The internal transcribed spacer (ITS) region and genes encoding Heat Shock Protein 60 (*HSP60*), the second-largest subunit of RNA polymerase II (*RPB2*), and glyceraldehyde 3-phosphate dehydrogenase (*G3PDH*) were partially amplified using the respective primer pairs ITS1/ITS2, HSP60f/HSP60r, RPB2f/RPB2r, and G3PDH-F/G3PDH-R (Staats et al., 2005; White et al., 1990). Phylogenetic trees were generated from concatenated sequences using the maximum likelihood (ML) and Bayesian inference (BI) methods in IQ-TREE 2.0.6 software (Nguyen et al., 2015; Minh et al., 2020; Jiang et al., 2022). The purified strains were inoculated on PDA plates and cultured at 25 °C under 75% relative humidity in the dark for 7–11 days to observe their morphological characteristics.

### 2.3 Fungal genome analysis

Genomic sequencing of HM-Y1 was performed to identify pathogenic fungi and characterize their genomes. Fungal mycelium of HM-Y1 cultured in potato dextrose broth (PDB) with shaking for 72 h was collected (Klug et al., 2024). DNA concentration, quality, and integrity were determined using a Qubit fluorometer (Invitrogen, United States) and a NanoDrop spectrophotometer (Thermo Scientific, United States). Sequencing libraries were constructed using the TruSeq DNA Sample Preparation Kit (Illumina, United States) and the Template Preparation Kit (Pacific Biosciences, United States). The genome of the pathogenic fungal strain HM-Y1 was sequenced using the Illumina MiSeq II and PacBio RS III sequencing platforms (Li Y. et al., 2025). The virulence factors of strain HM-Y1 were characterized using the Comprehensive Antibiotic Resistance Database (CARD), the Carbohydrate-Active Enzymes Database (CAZy), the Database of Fungal Virulence Factors (DFVF), Gene Ontology (GO) analysis, Kyoto Encyclopedia of Genes and Genomes (KEGG) analysis, the Evolutionary Genealogy of Genes: Non-supervised Orthologous Groups (eggNOG) database, the Non-Redundant (NR) protein database, Cytochrome P450 (CYP) analysis, the Pathogen–Host Interactions database (PHI), secondary metabolite analysis, and the Transporter Classification Database (TCDB) (Chen et al., 2026; Qiu et al., 2026; Yan et al., 2026; Shi et al., 2024).

### 2.4 Sensitivity of the pathogen HM-Y1 to pydiflumetofen

HM-Y1 mycelial plugs (5 mm in diameter) were excised from the margins of 3-day-old colonies grown on PDA and placed upside down in the center of 9-cm Petri dishes containing serial concentrations (0, 0.05, 0.10, 0.25, 0.50, and 1.00 mg/L) of pydiflumetofen (98% available ingredient; Syngenta (China) Investment Co., Ltd.) (He et al., 2020; Li et al., 2022; Zhou et al., 2024). Cultures were incubated at 25 °C in the dark for 5 days (He et al., 2020; Zhao et al., 2022; Chen et al., 2024). Colony diameters were measured using the cross method and expressed as a percentage of growth inhibition. Each treatment was replicated three times, and the experiment was repeated twice (Li et al., 2022; Zhao et al., 2022).

### 2.5 Pydiflumetofen efficacy on postharvest strawberry rot

After preliminary experiments, five strawberry fruits were sprayed with pydiflumetofen (200 g/L SC; Syngenta (China) Investment Co. Ltd.) at concentrations of 2 mg/L. After HM-Y1 inoculation, all fruits were covered with plastic bags for 12 h, after which the bags were removed. Disease severity was assessed 5 d after inoculation by observing the symptoms exhibited by the fruits. All experiments were repeated three times. The natural decay incidence of strawberries was calculated using the following formula: number of rotten strawberries/total number of strawberries × 100% (Zhao et al., 2023). Control efficiency (%) was calculated as follows: (incidence rate of control group − incidence rate of treatment group) / incidence rate of control group × 100.

### 2.6 Data analysis

The EC_50_ values for each isolate were determined by linear regression analysis of the inhibition rate versus the logarithm (log10) of fungicide concentration (Li J. et al., 2025). Regression equations of fungal mycelial growth inhibition were calculated using SPSS 26.0. Significant differences among treatments were determined using Duncan’s new complex range method and were plotted using Origin 10.0.

## 3 Results

### 3.1 Fungal and bacterial communities of healthy and diseased strawberry fruits

**Figure 1:**
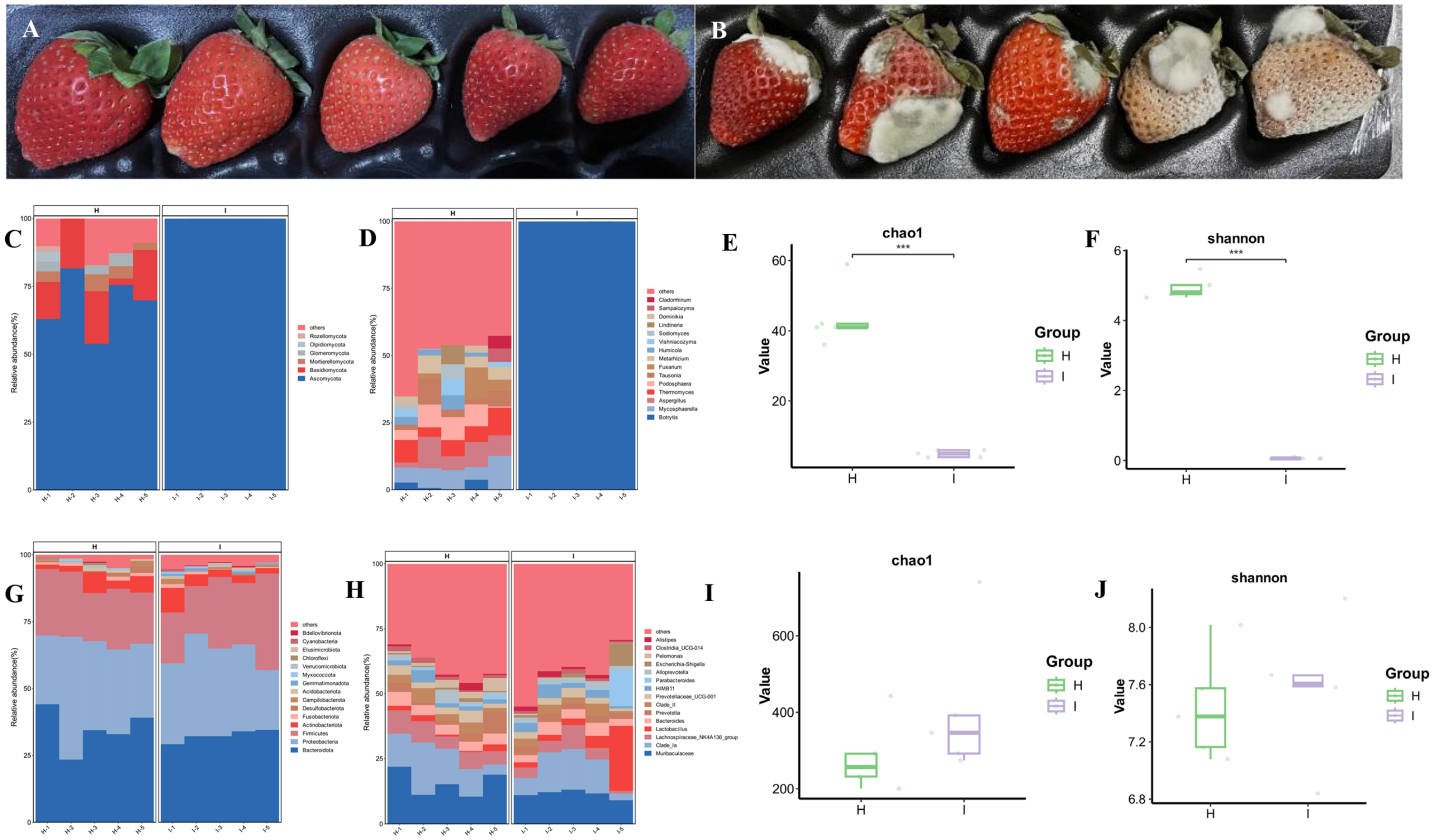
Microbial community of strawberry fruits. **(A)** Collected healthy strawberry fruit; **(B)** Typical symptoms of postharvest fruit rot on collected strawberry fruit; Phylum **(C)**, genus **(D)**, Chao1 **(E)**, and Shannon **(F)** of fungal communities of healthy and diseased strawberry fruits; Phylum **(G)**, genus **(H)**, Chao1 **(I)**, and Shannon **(J)** of bacterial communities of healthy and diseased strawberry fruits. Group H: healthy (unrotten) strawberry fruits; Group I: infected by pathogen (rotten) strawberry fruits.

Compared with the strawberries in group H, those in group I showed complete rotting (Figures 1A,B). The high-throughput analysis of the fungal community in strawberry fruits revealed that Ascomycota at the phylum level (Figure 1C) and *Botrytis* at the genus level (Figure 1D) were present only in group I. Other fungal phyla and genera were present in group H; Rozellomycota, Olpidiomycota, Glomeromycota, Mortierellomycota, and Basidiomycota (Figure 1C) and *Cladorrhinum*, *Sampaiozyma*, *Dominikia*, *Lindtneria*, *Sodiomyces*, *Vishniacozyma*, *Humicola*, *Metarhizium*, *Fusarium*, *Tausonia*, *Podosphaera*, *Thermomyces*, *Aspergillus*, and *Mycosphaerella* (Figure 1D), respectively. The Chao1 (Figure 1E) and Shannon (Figure 1F) indices of the fungal communities exhibited significant differences.

The phyla Bdellovibrionota, Cyanobacteria, Elusimicrobiota, Chloroflexi, Verrucomicrobiota, Myxococcota, Gemmatimonadota, Acidobacteriota, Campilobacterota, Desulfobacterota, Fusobacteriota, Actinobacteriota, Firmicutes, Proteobacteria, and Bacteroidota were present in both group I and group H (Figure 1G). At the genus level, *Alistipes*, *Clostridia*, *Pelomonas*, *Escherichia-Shigella*, *Alloprevotella*, *Parabacteroides HIMB11*, *Prevotellaceae UCG-001*, *Cladell*, *Prevotella*, *Bacteroides*, *Actobacilus*, *Lachnospiraceae* NK4A136, *Cladela*, and *Vuribaculaceae* were also present in both groups (Figure 1H). The Chao1 (Figure 1I) and Shannon (Figure 1J) indices of the fungal communities showed no significant differences.

### 3.2 Isolation and identification of *Botrytis cinerea* HM-Y1

**Figure 2:**
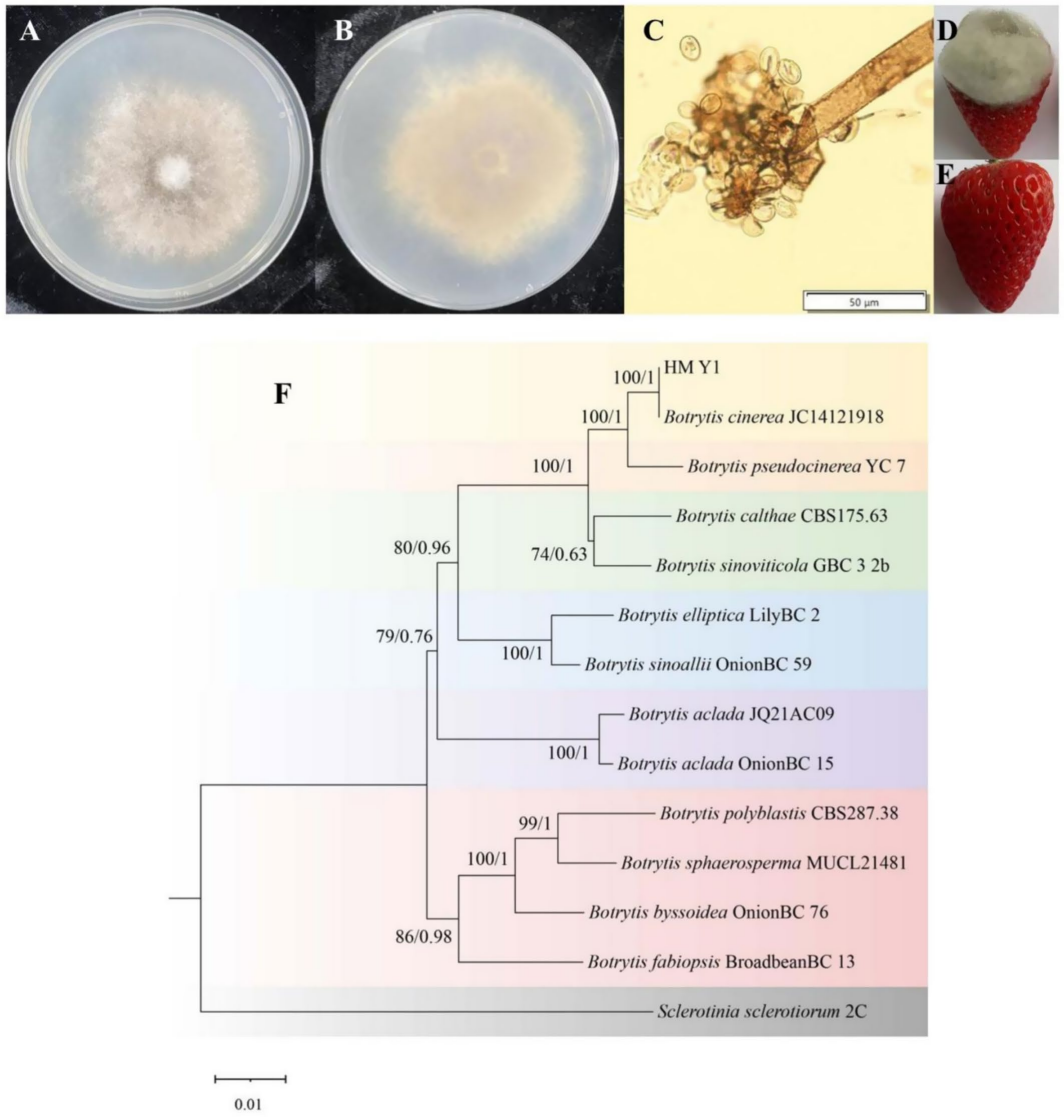
Morphological characteristics of isolate HM-Y1 causing strawberry fruit rot. Front **(A)** and back **(B)** views of *B. cinerea* fungal HM-Y1 colonies isolated from infected strawberry fruit; **(C)** Conidiophores bearing conidia of *B. cinerea* HM-Y1. Symptoms of strawberry fruit after inoculation with *B. cinerea* HM-Y1 **(D)** and clean water **(E)**; **(F)** Phylogenetic tree of the isolate HM-Y1 based on combined rDNA-ITS, HSP60, RPB2, and G3PDH gene sequences.

The HM-Y1 strain was purified, yielding dark brown colonies on PDA plates after 5 d at 25 °C (Figures 2A,B). The conidia (*n* = 50) were single-celled, hyaline, either ellipsoid or ovoid, and measured 7.2–15.3 × 5.7–11.7 μm (Figure 2C). The conidiophores were branched at the apex, bearing bunches of conidia resembling grape clusters. The sequences of the *ITS* (GenBank PX455751), *HSP60* (GenBank PX515975), *RPB2* (GenBank PX515976), and *G3PDH* (GenBank PX515977) genes showed 100% homology with the sequences of the *Botrytis cinerea* isolate. Five strawberry fruits inoculated with isolate HM-Y1 displayed fruit rot symptoms (Figures 2D,E). This isolate was reisolated from infected fruits and showed the same morphological and molecular traits, confirming Koch’s postulates and verifying its identification as *B. cinerea*. Thus, the isolate HM-Y1 was identified as *B. cinerea* according to its morphology and molecular characteristics (Figure 2F).

### 3.3 Genomic analysis of *Botrytis cinerea* HM-Y1

**Figure 3:**
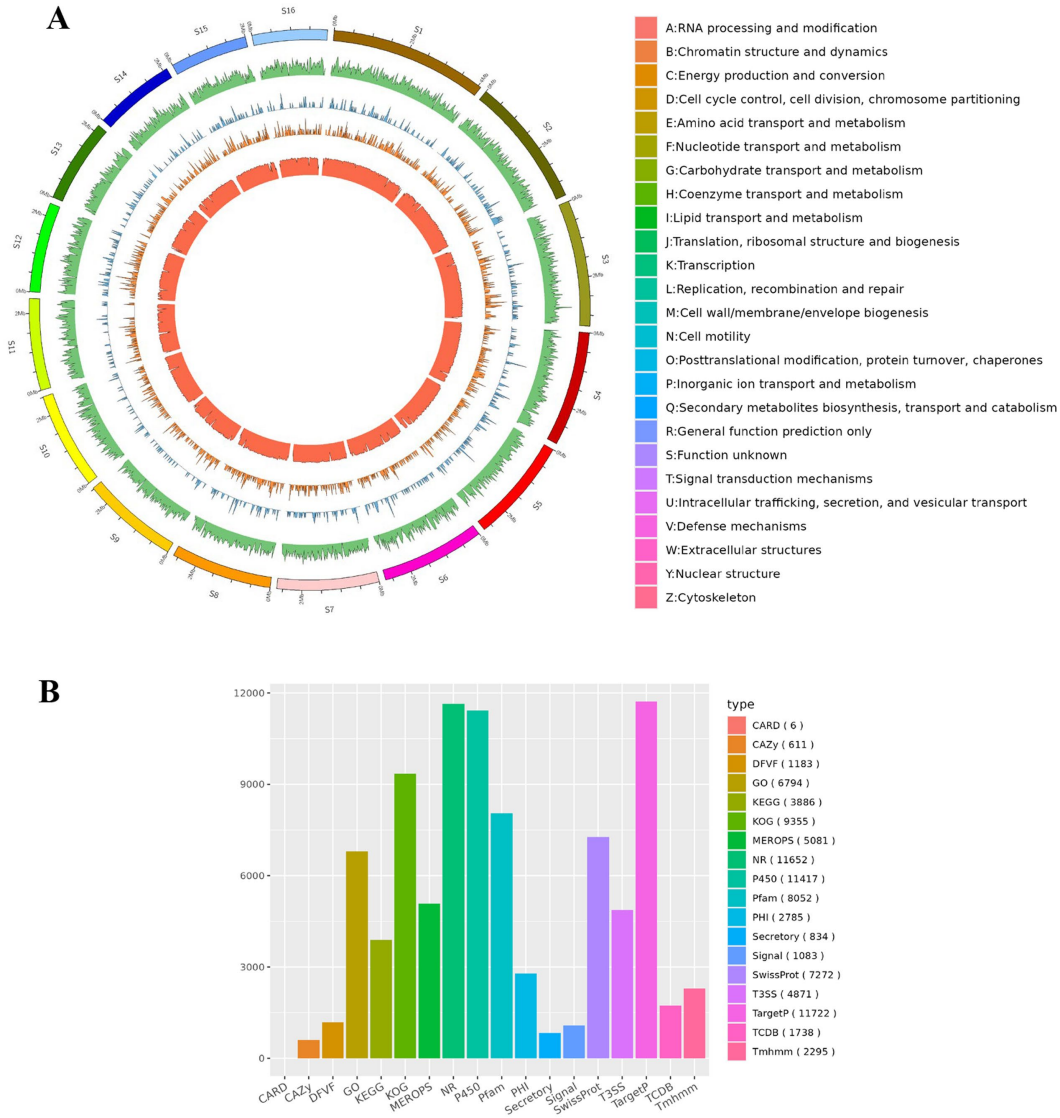
HM-Y1 genomic circle map **(A)** and statistical chart of protein-coding gene function annotation **(B)**. Based on the results of gene prediction and annotation information, the CIROS software was used to draw the loop map of the genome. By default, sequences longer than 1 M in length were selected for drawing; From the inside out: circle 1: collinearity; Circle 2: G + C content; Circle 3: DFVF; Lap 4: CAZy; Circle 5: gene density.

Genomic analysis (GenBank JBXSQN000000000) revealed the minimum, maximum, and total sequence length (bp) of *Botrytis cinerea* HM-Y1 to be 9,372, 4,076,437, 43,237,401, respectively, with a GC content of 41.95% and an N50 contig length of 2,648,912 bp (Figure 3A). It generated a high-quality assembly with 98.5% completeness as assessed by BUSCO. The copy numbers of ncRNA, 5S rRNA, 5.8S rRNA, 18S rRNA, 28S rRNA, and tRNA were found to be 55, 1, 2, 4, 2, and 231, respectively. The total lengths (bp) of ncRNA, 5S rRNA, 5.8S rRNA, 18S rRNA, 28S rRNA, and tRNA were 7,888, 304, 5,835, 7,294, and 21,563, respectively. A statistical chart of protein-coding gene function annotation (Figure 3B) was used for CARD (6), CAZy (611), DFVF (1183), GO (6794), KEGG (3886), KOG (9355), MEROPS (5081), NR (11652), P450 (11417), Pfam (8052), PHI (2785), Secretory (834), Signal (1083), SwissProt (7272), T3SS (4871), TargetP (11722), TCDB (1738), and Tmhmm (2295) analysis.

#### 3.3.1 CARD analysis of *Botrytis cinerea* HM-Y1

In CARD, the number of genes in the “Antibiotic Resistance,” “Antibiotic Target,” and “Antibiotic Biosynthesis” categories were 4, 1, and 1, respectively (Table 1). The four genes in the “Antibiotic Resistance” category were scaffold12.t411, scaffold12.t632, scaffold3.t215, and scaffold5.t68, closely related to thymidylate synthase, APH(3″)-Ib Curated, elongation factor Tu, and Monooxygenase EthA, respectively. The gene in the “Antibiotic Target” category was scaffold5.t262, closely related to translation elongation factor G; that in the “Antibiotic Biosynthesis” category was scaffold11.t351, closely related to acetolactate synthase catalytic subunit.

**Table 1:** Antibiotic resistance analysis statistics in CARD.

Property	Number of genes	Percentage (%)	Query_name
Antibiotic resistance	4	0.03	scaffold12.t411, scaffold12.t632, scaffold3.t215, and scaffold5.t68
Antibiotic target	1	0.01	scaffold5.t262
Antibiotic biosynthesis	1	0.01	scaffold11.t351

#### 3.3.2 CAZy analysis of *Botrytis cinerea* HM-Y1

**Figure 4:**
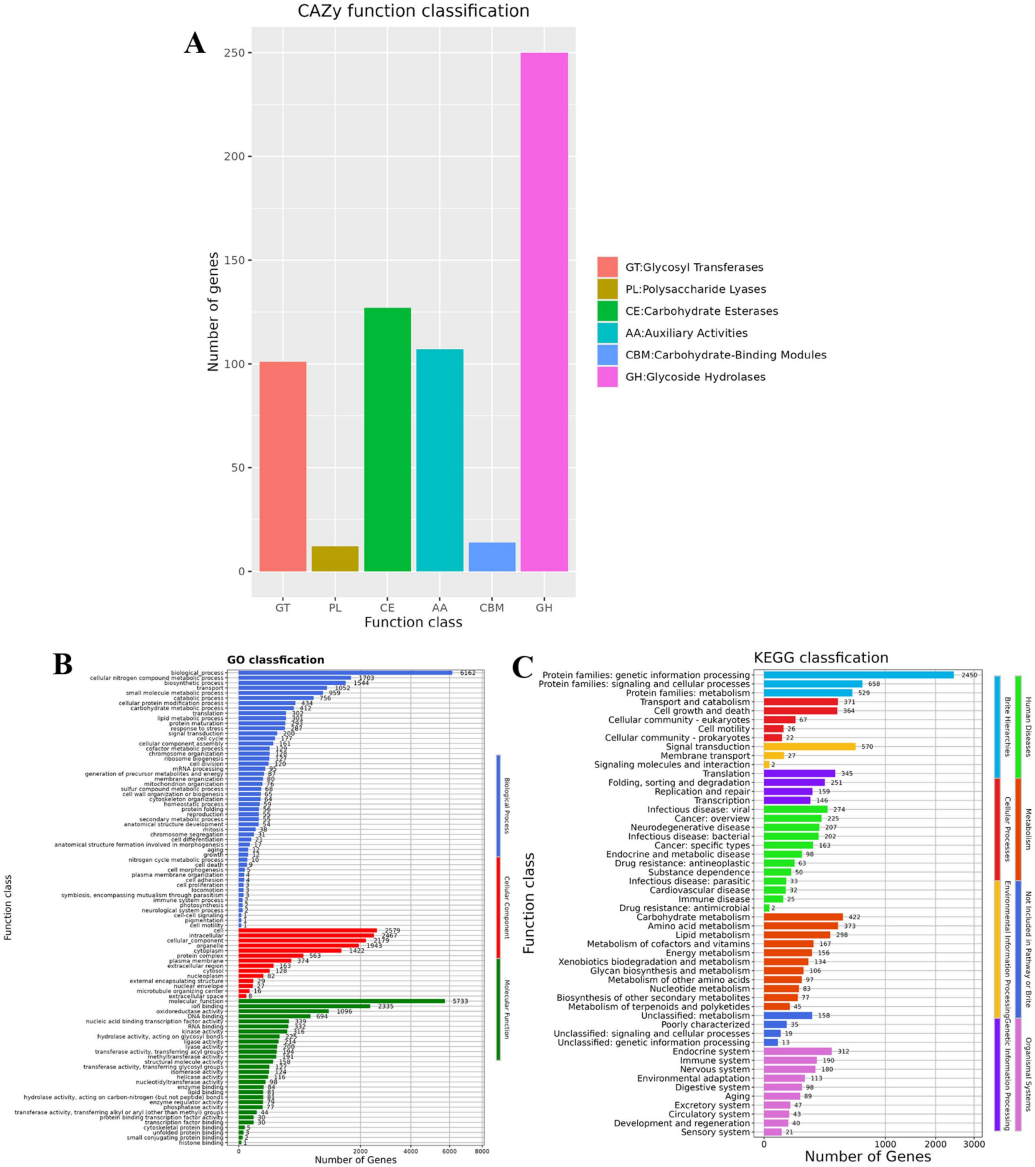
CAZy **(A)**, GO **(B)**, and KEGG **(C)** function classification diagram.

The CAZy database analysis (Figure 4A) revealed 101 glycosyl transferases (GTs), 12 polysaccharide lyases (PLs), 127 carbohydrate esterases (CEs), 107 auxiliary activities (AAs), 14 carbohydrate-binding modules (CBMs), and 250 glycoside hydrolases (GHs), accounting for 0.8616, 0.1024, 1.0834, 0.9128, 0.1194, and 2.1327%, respectively.

#### 3.3.3 DFVF analysis of *Botrytis cinerea* HM-Y1

In the DFVF analysis (Table 2), the annotated function of Hit_name BOT2_BOTFU was identified as presilphiperfolan-8-beta-ol synthase, which catalyzes the cyclization of farnesyl diphosphate (FPP) to presilphiperfolan-8-beta-ol (PSP), representing the committed step in the biosynthesis of the terpenoid virulence factor botrydial (Li S. et al., 2025). This enzyme exhibits the following catalytic activity: farnesyl diphosphate + H(2)O = presilphiperfolan-8-beta-ol + diphosphate, requiring three magnesium ions per subunit as cofactors (by similarity). Regarding biophysiochemical properties, the enzyme exhibits a Km value of 6.04 μM for farnesyl diphosphate. Expression of the encoding gene is downregulated by the calcineurin inhibitor cyclosporin A and by BCG1 inactivation. Regarding domains, the enzyme exhibits the Asp-Asp-Xaa-Xaa-Asp/Glu (DDXXD/E) motif, which is important for its catalytic activity, presumably by binding Mg(2+) (by similarity). Furthermore, the BOT2-encoding gene belongs to the botrydial biosynthetic gene cluster, comprising 5 genes (*BOT1*–*BOT5*) involved in botrydial synthesis and coregulated by the BCG1–calcineurin transduction pathway. Finally, the similarity analysis revealed that the enzyme belongs to the terpene synthase family.

**Table 2:** Analysis of Botrydial synthesis, ABC and MFS transporters in DFVF.

Function	Hit_name	Hit_length	Hit_start	Hit_end	Aln_length	Identity	*E*-value
Botrydial synthesis	BOT2_BOTFU	399	1	399	399	100	1.87e-299
ABC transporter superfamily.	Q3Y5V5_MAGGR	1,321	25	1,321	1,342	46.50	0
	Q3Y5V5_MAGGR	1,321	762	1,298	551	28.68	2.38e-51
	Q874F3_MAGGR	1,484	49	1,484	1,446	65.49	0
	Q3Y5V5_MAGGR	1,321	26	1,310	1,349	30.39	4.23e-161
	O13407_MAGGR	1,619	103	1,542	1,447	60.06	0
	Q5A762_CANAL	1,606	736	1,594	885	29.83	3.32e-94
	Q9UW03_BOTFU	1,439	61	1,439	1,385	64.98	0
	Q96VL9_BOTFU	1,501	1,090	1,252	163	41.72	6.75e-30
	O60034_BOTFU	1,562	1	1,562	1,565	95.97	0
	Q3Y5V5_MAGGR	1,321	373	619	247	38.46	1.74e-46
	Q3Y5V5_MAGGR	1,321	32	1,318	1,290	33.64	6.66e-232
	Q3Y5V5_MAGGR	1,321	38	1,319	1,312	30.64	1.08e-151
	Q9UW03_BOTFU	1,439	1	1,439	1,439	99.37	0
	Q874F3_MAGGR	1,484	834	1,140	328	32.32	4.66e-32
	O60034_BOTFU	1,562	213	758	557	22.26	3.52e-37
	Q96VL9_BOTFU	1,501	1	1,501	1,501	99.93	0
	Q96VL9_BOTFU	1,501	78	1,490	1,425	50.25	0
	Q9UW87_CANAL	1,606	1,279	1,603	332	29.82	3.23e-35
	Q3Y5V5_MAGGR	1,321	1,070	1,317	251	40.24	1.27e-49
	Q3Y5V5_MAGGR	1,321	37	1,319	1,340	30.52	2.45e-157
	Q5A762_CANAL	1,606	316	1,601	1,320	25.61	8.14e-87
	Q3Y5V5_MAGGR	1,321	114	629	519	30.44	8.51e-50
	Q874F3_MAGGR	1,484	872	1,366	516	31.01	1.68e-44
	Q5A762_CANAL	1,606	286	901	641	23.87	1.92e-38
	Q5A762_CANAL	1,606	378	1,606	1,312	29.95	5.98e-141
	O60034_BOTFU	1,562	158	1,514	1,389	35.93	1.47e-256
	Q3Y5V5_MAGGR	1,321	34	1,321	1,309	33.08	1.11e-208
	Q5A762_CANAL	1,606	449	1,603	1,207	31.57	5.99e-154
	Q5A762_CANAL	1,606	473	1,604	1,163	26.83	1.65e-92
	Q9UW87_CANAL	1,606	689	1,580	931	25.67	2.01e-70
	Q5A762_CANAL	1,606	294	1,603	1,435	29.69	2.94e-152
	Q5A762_CANAL	1,606	758	1,581	837	28.79	7.53e-87
	Q5A762_CANAL	1,606	193	1,603	1,491	29.64	7.14e-146
	Q5A762_CANAL	1,606	500	1,442	983	25.03	3.83e-55
	Q3Y5V5_MAGGR	1,321	365	619	255	41.18	5.27e-48
	Q5A762_CANAL	1,606	652	1,602	975	26.97	5.48e-93
	Q3Y5V5_MAGGR	1,321	15	1,321	1,313	52.55	0
	Q9C1I7_MYCGR	1,635	97	1,533	1,463	59.67	0
	Q5A762_CANAL	1,606	473	1,603	1,185	30.72	8.24e-134
	Q3Y5V5_MAGGR	1,321	32	1,321	1,307	37.26	7.28e-246
	Q9UW87_CANAL	1,606	22	1,603	1,621	36.52	2.96e-292
Major facilitator superfamily	Q5ANE1_CANAL	748	43	496	491	32.18	1.13e-60
	Q5XTQ5_BOTFU	615	104	559	482	24.27	1.51e-23
	Q5ANE1_CANAL	748	33	506	488	29.71	1.76e-53
	Q5XTQ5_BOTFU	615	112	544	452	23.45	1.61e-22
	Q5XTQ5_BOTFU	615	133	569	447	22.82	1.68e-23
	Q5XTQ5_BOTFU	615	144	550	424	23.58	1.21e-12
	Q5ANE1_CANAL	748	122	496	386	28.50	1.21e-38
	Q5XTQ5_BOTFU	615	103	550	486	25.93	2.29e-32
	Q5ANE1_CANAL	748	35	509	499	39.48	4.52e-103
	Q5ANE1_CANAL	748	35	495	485	42.06	1.09e-119
	Q5XTQ5_BOTFU	615	104	548	469	26.01	6.85e-31
	Q5XTQ5_BOTFU	615	98	551	494	22.87	3.43e-25
	Q5XTQ5_BOTFU	615	5	564	577	28.08	1.42e-57
	Q5XTQ5_BOTFU	615	90	548	499	27.66	7.80e-41
	Q5XTQ5_BOTFU	615	104	553	487	27.10	1.37e-34
	Q5ANE1_CANAL	748	31	498	479	28.60	1.30e-51
	Q5XTQ5_BOTFU	615	72	552	500	30.2	1.04e-61
	Q5XTQ5_BOTFU	615	20	552	559	27.01	2.23e-50
	Q5ANE1_CANAL	748	40	496	465	27.10	2.04e-44
	Q5XTQ5_BOTFU	615	99	552	469	27.29	1.18e-38
	Q5XTQ5_BOTFU	615	101	548	464	23.49	4.12e-24
	Q5XTQ5_BOTFU	615	93	554	479	25.26	4.79e-22
	Q5XTQ5_BOTFU	615	103	552	468	29.06	7.78e-47
	Q5XTQ5_BOTFU	615	98	593	556	25.18	5.12e-27
	Q5XTQ5_BOTFU	615	149	550	416	22.36	3.24e-17
	Q5XTQ5_BOTFU	615	102	577	509	25.93	6.36e-32
	Q5ANE1_CANAL	748	32	512	492	42.48	1.31e-126
	Q5ANE1_CANAL	748	23	508	495	40.61	2.70e-119
	Q5ANE1_CANAL	748	39	513	486	27.78	7.32e-39
	Q5XTQ5_BOTFU	615	101	548	508	28.94	1.32e-49
	Q5XTQ5_BOTFU	615	110	548	459	28.32	1.55e-36
	Q5ANE1_CANAL	748	44	495	461	38.61	4.71e-103
	Q5XTQ5_BOTFU	615	105	569	498	23.69	2.41e-28
	Q5XTQ5_BOTFU	615	98	569	500	29.6	1.18e-37
	Q5ANE1_CANAL	748	49	501	472	30.08	1.32e-49
	Q5XTQ5_BOTFU	615	1	615	615	100	0
	Q5ANE1_CANAL	748	39	511	499	30.06	9.35e-68
	Q5XTQ5_BOTFU	615	104	550	471	27.18	2.75e-34
	Q5ANE1_CANAL	748	43	496	471	33.12	9.70e-59
	Q5ANE1_CANAL	748	24	497	502	32.27	7.03e-70
	Q5XTQ5_BOTFU	615	105	498	425	26.12	8.74e-22
	Q5XTQ5_BOTFU	615	103	556	479	25.89	1.57e-29
	Q5ANE1_CANAL	748	36	541	526	41.44	3.26e-137
	Q5ANE1_CANAL	748	37	496	492	31.30	9.00e-73

The 41st and 44th Hit_name of *B. cinerea* HM-Y1 were found to be ATP-binding cassette (ABC) and major facilitator superfamily (MFS) transporters (Table 2), which dispose of key phytoalexins by exporting them from fungal cells.

#### 3.3.4 GO analysis of *Botrytis cinerea* HM-Y1

In the GO analysis, *B. cinerea* HM-Y1 was found to participate in biological processes, molecular functions, and cellular components (Figure 4B); notably, the biological processes “toxin biosynthetic process” (GO:0009403) and “toxin metabolic process” (GO:0009404) (Table 3).

**Table 3:** Toxin-related processes in GO.

protein_id	GO_number	GO_function	GO_ontologies
scaffold12.t662	GO:0009403	Toxin biosynthetic process	Biological process
scaffold12.t662	GO:0009404	Toxin metabolic process	Biological process
scaffold14.t419	GO:0009403	Toxin biosynthetic process	Biological process
scaffold14.t419	GO:0009404	Toxin metabolic process	Biological process
scaffold4.t631	GO:0009403	Toxin biosynthetic process	Biological process
scaffold4.t631	GO:0009404	Toxin metabolic process	Biological process
scaffold7.t168	GO:0009403	Toxin biosynthetic process	Biological process
scaffold7.t168	GO:0009404	Toxin metabolic process	Biological process
scaffold9.t7	GO:0009403	Toxin biosynthetic process	Biological process
scaffold9.t7	GO:0009404	Toxin metabolic process	Biological process

#### 3.3.5 KEGG analysis of *Botrytis cinerea* HM-Y1

In the KEGG analysis (Figure 4C), 2,450 genes were annotated to “Protein families: genetic information processing” in Brite Hierarchies, 371 to “Transport and catabolism” in “Cellular Processes,” 570 to “Signal transduction” in “Environmental Information Processing,” 345 to “Translation” in “Genetic Information Processing,” 422 to “Carbohydrate metabolism” in “Metabolism,” 158 to “Unclassified: metabolism” in “Unclassified: metabolism,” and 312 to “Endocrine system” in “Organismal Systems.” The pathogenic fungus *B. cinerea* HM-Y1 constantly exchanges substances and energy, maintains its own activities, and responds to the external environment, enabling strong environmental adaptability. In addition, the strain exhibited abundant secondary metabolite biosynthesis genes, with metabolic pathways closely related to cellular gene function.

#### 3.3.6 eggNOG analysis of *Botrytis cinerea* HM-Y1

**Figure 5:**
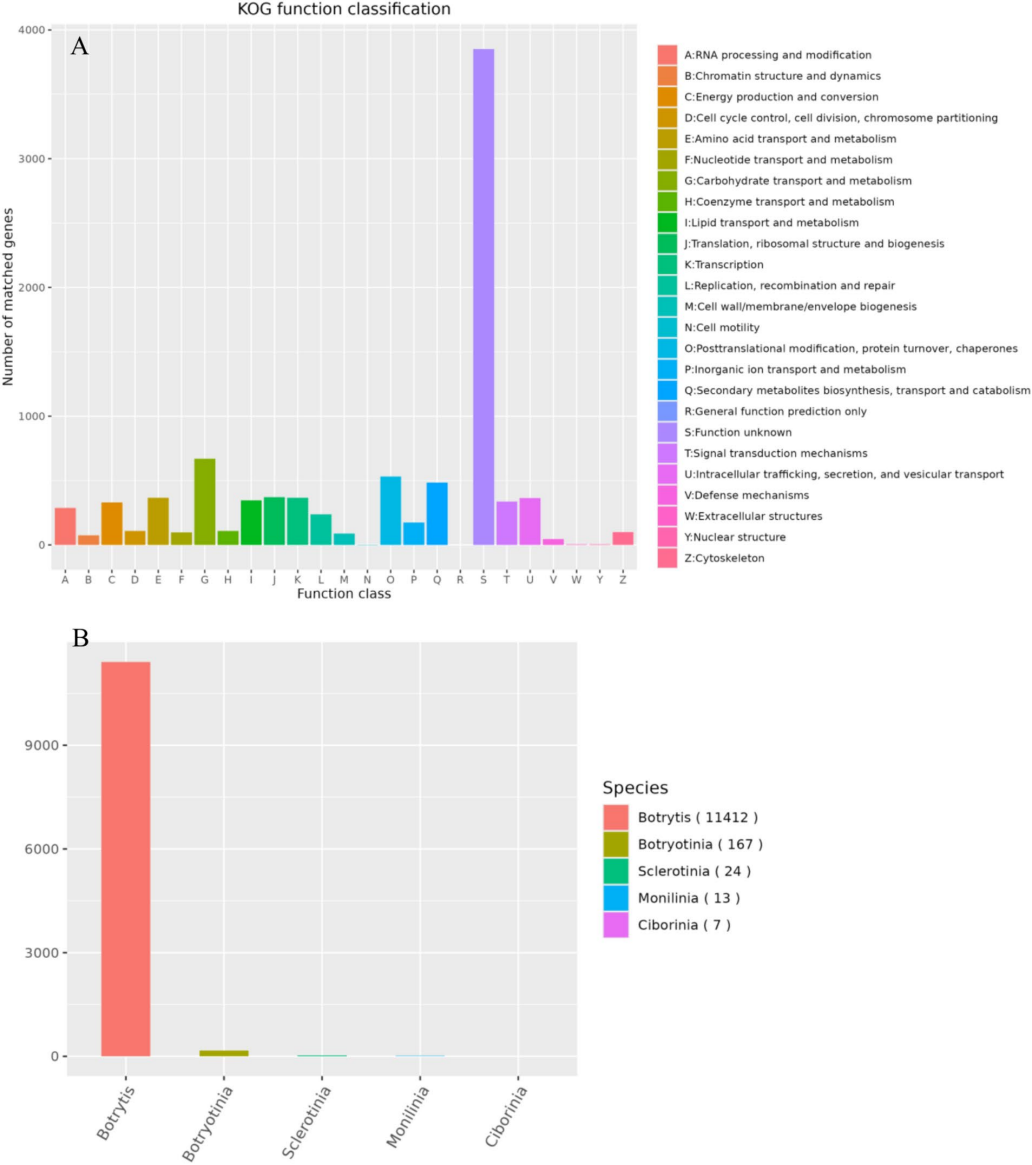
EggNOG **(A)** and NR **(B)** function classification.

In the eggNOG analysis, 9,355 genes were annotated into 25 functional categories (Figure 5A). The largest category, containing 3,852 genes, represented genes of unknown function, accounting for 32.86%, indicating that the gene functions of *B. cinerea* HM-Y1 can still be further classified and analyzed. The category with the second-highest number of genes (669) was “Carbohydrate transport and metabolism,” accounting for 5.71%.

#### 3.3.7 NR analysis of *Botrytis cinerea* HM-Y1

Strain HM-Y1 exhibited the highest similarity to the *Botrytis* genus in the NR analysis (Figure 5B). Additionally, protein-coding gene sequences of 167, 24, 13, and 7 amino acids in length exhibited high similarity to the *Botryotinia*, *Sclerotinia*, *Monilinia*, and *Ciborinia* genera.

#### 3.3.8 CYP analysis of *Botrytis cinerea* HM-Y1

The CYP analysis revealed that *bcbot1* encodes a P450 monooxygenase inferred from genomic data (Coca-Ruiz et al., 2024), providing evidence that it is involved in the botrydial pathway (Table 4). *Bcaba1* represents the first identified fungal abscisic acid (ABA) biosynthetic gene (Liu et al., 2024; Wang et al., 2024), demonstrating that the phytopathogenic ascomycete *B. cinerea* HM-Y1 maybe produce ABA, which is thought to be involved in host–pathogen interactions (Table 4).

**Table 4:** Analysis of CYP51 and Bccpr1 in CYP.

P450	Function	Hit_length	Hit_start	Hit_end	Aln_length	Identity	*E*-value
BC1G_11853	CYP51; Eburicol 14-alpha-demethylase	522	1	522	522	100	0
gm1.7036_g	Bccpr1; putative nadph-cytochrome p450 reductase protein; NADPH cytochrome P450 oxidoreductase	1,057	463	514	52	34.62	2.8

#### 3.3.9 PHI analysis of *Botrytis cinerea* HM-Y1

The PHI analysis revealed 1,351 genes that cause loss of pathogenicity, 1,233 that do not affect pathogenicity, and 99 that increase pathogenicity (hypervirulence). Moreover, 14 genes were identified (Table 5) that determine plant virulence and can directly or indirectly enable the host to identify the pathogen and activate plant defense responses.

**Table 5:** The gene of determine plant virulence through PHI.

Hit_name	Hit_description	Hit_length	Hit_start	Hit_end	Aln_length	Identity	*E*-value
Q6ZX14	PHI:325	4,034	11	3,950	4,085	35.18	0
D2TI55	PHI:9334	264	3	255	256	42.19	3.56e-65
Q6ZX14	PHI:325	4,034	11	2,493	2,538	36.13	0
G4ZHR2	PHI:6868	241	5	240	239	53.56	1.22e-72
A0A059UDR8	PHI:2897	316	40	316	291	46.39	6.79e-92
G4MVX4	PHI:3216	295	1	238	244	43.44	2.01e-49
A0A0A2ILW0	PHI:7664	422	1	246	253	51.38	1.15e-72
Q8RP09	PHI:981	432	359	421	63	46.03	4.49e-09
Q5ZVD8	PHI:6520	479	13	466	459	40.52	2.04e-108
G4ZHR2	PHI:6868	241	13	241	230	52.17	6.18e-70
A0A0A2ILW0	PHI:7664	422	48	224	192	41.15	8.92e-27
A0A0A2ILW0	PHI:7664	422	38	231	208	49.04	3.35e-56
A0N0D1	PHI:2216	316	1	248	271	49.82	1.45e-48
Q6ZX14	PHI:325	4,034	3,330	4,031	751	33.16	1.57e-99

#### 3.3.10 Secondary metabolite analysis of *Botrytis cinerea* HM-Y1

The secondary metabolite gene cluster analysis (Table 6) revealed that *B. cinerea* HM-Y1 produce NRPS, T1PKS, T3PKS, fungal-RiPP-like, terpene, NAPAA, and indole. The following were identified in the most similar known cluster: metachelin C; metachelin A; metachelin A–CE; metachelin B; dimerumic acid 11-mannoside; dimerumic acid (NRP); botcinic acid (polyketide); 1,3,6,8-tetrahydroxynaphthalene (polyketide); scytalone; T3HN (polyketide); citrinin (iterative type I polyketide); squalestatin S1 (terpene); aspulvinone H; aspulvinone B1 (NRP); ACT-toxin II (polyketide); botcinin H; botcinin I; botcinin J; cinbotolide D; botcinin K (terpene); fusaristatin A (NRP–polyketide hybrid); and alternariol (polyketide).

**Table 6:** Analysis of secondary metabolite gene clusters.

Region	Type	From	To	Most similar known cluster
Region1.1	NRPS, T1PKS	9,859	90,260	–
Region1.2	Fungal-RiPP-like	2,297,321	2,388,346	–
Region1.3	Terpene	2,417,402	2,448,598	–
Region1.4	NRPS	2,680,532	2,747,137	Metachelin C; metachelin A; metachelin A-CE; metachelin B; dimerumic acid 11-mannoside; dimerumic acid NRP
Region1.5	Terpene	2,768,560	2,799,646	–
Region1.6	T1PKS	4,000,840	4,076,437	Botcinic acid Polyketide
Region2.1	Fungal-RiPP-like, T1PKS	190,689	319,377	1,3,6,8-tetrahydroxynaphthalene Polyketide
Region2.2	Fungal-RiPP-like	692,787	782,529	–
Region2.3	Fungal-RiPP-like	1,670,857	1,752,094	–
Region2.4	NRPS	2,505,739	2,574,466	–
Region2.5	T1PKS	2,730,900	2,800,785	–
Region2.6	Fungal-RiPP-like	3,004,273	3,095,401	–
Region3.1	T1PKS	447,887	507,918	Scytalone; T3HN Polyketide
Region3.2	NRPS, T1PKS	1,769,585	1,841,699	–
Region3.3	T1PKS, fungal-RiPP-like	2,556,451	2,653,629	Citrinin Polyketide: Iterative type I polyketide
Region3.4	NRPS-like	2,705,042	2,773,143	–
Region3.5	NRPS-like	3,137,985	3,206,097	–
Region4.1	Terpene	1,999,447	2,030,572	–
Region4.2	T1PKS	2,129,749	2,195,549	–
Region4.3	T1PKS	2,901,918	2,970,099	–
Region5.1	Terpene	804,724	836,326	Squalestatin S1 Terpene
Region5.2	other, fungal-RiPP-like, NRPS-like	839,229	943,132	–
Region5.3	NRPS	1,449,845	1,513,593	–
Region6.1	Terpene	1,711,527	1,742,516	–
Region6.2	T1PKS	2,512,581	2,579,019	–
Region7.1	Fungal-RiPP-like	134,842	223,153	–
Region7.2	NAPAA	493,653	543,924	NRP
Region7.3	T1PKS, NRPS-like	1,535,962	1,641,889	–
Region7.4	Fungal-RiPP-like, NRPS-like	2,164,633	2,281,958	Aspulvinone H; aspulvinone B1 NRP
Region8.1	NRPS-like	754,334	817,887	–
Region9.1	NRPS-like, T1PKS	2,410,811	2,483,811	ACT-Toxin II Polyketide
Region10.1	Fungal-RiPP-like	166,152	257,054	**–**
Region10.2	Terpene	1,184,938	1,216,417	**–**
Region10.3	NRPS-like	1,931,654	1,984,909	**–**
Region10.4	T1PKS	2,225,382	2,293,928	**–**
Region10.5	NRPS-like	2,410,865	2,474,362	**–**
Region11.1	Terpene	40,664	71,682	**–**
Region11.2	Fungal-RiPP-like	264,301	353,877	**–**
Region11.3	Fungal-RiPP-like	504,280	595,313	**–**
Region11.4	T1PKS, NRPS	1,384,616	1,465,841	**–**
Region12.1	Terpene	112,612	144,332	Botcinin H; botcinin I; botcinin J; cinbotolide D; botcinin K Terpene
Region12.2	Fungal-RiPP-like	198,411	289,531	–
Region12.3	NRPS-like, NRPS	603,553	693,313	–
Region12.4	NRP-metallophore, NRPS	2,039,944	2,125,878	–
Region13.1	Fungal-RiPP-like	392,209	483,242	–
Region13.2	T1PKS	534,758	602,424	–
Region13.3	T3PKS	729,054	790,766	–
Region14.1	Indole	256,517	288,309	–
Region14.2	NRPS, T1PKS, terpene	1,495,922	1,612,354	Fusaristatin A NRP + Polyketide
Region14.3	T1PKS	1,828,257	1,896,895	–
Region15.1	NRPS-like	283,878	341,223	–
Region15.2	Fungal-RiPP-like	596,541	687,404	–
Region15.3	NRPS-like	935,454	998,967	–
Region15.4	Fungal-RiPP-like	1,055,744	1,143,374	–
Region15.5	Fungal-RiPP-like	1,310,575	1,402,600	–
Region16.1	T1PKS	105,242	169,397	–
Region16.2	NRPS	607,251	681,384	–
Region16.3	Fungal-RiPP-like	825,324	913,077	–
Region16.4	Indole, fungal-RiPP-like, T1PKS	1,200,362	1,305,331	Alternariol Polyketide
Region16.5	Fungal-RiPP-like	1,794,619	1,889,495	–
Region18.1	NRPS-like	1	49,428	–

#### 3.3.11 TCDB analysis of *Botrytis cinerea* HM-Y1

**Figure 6:**
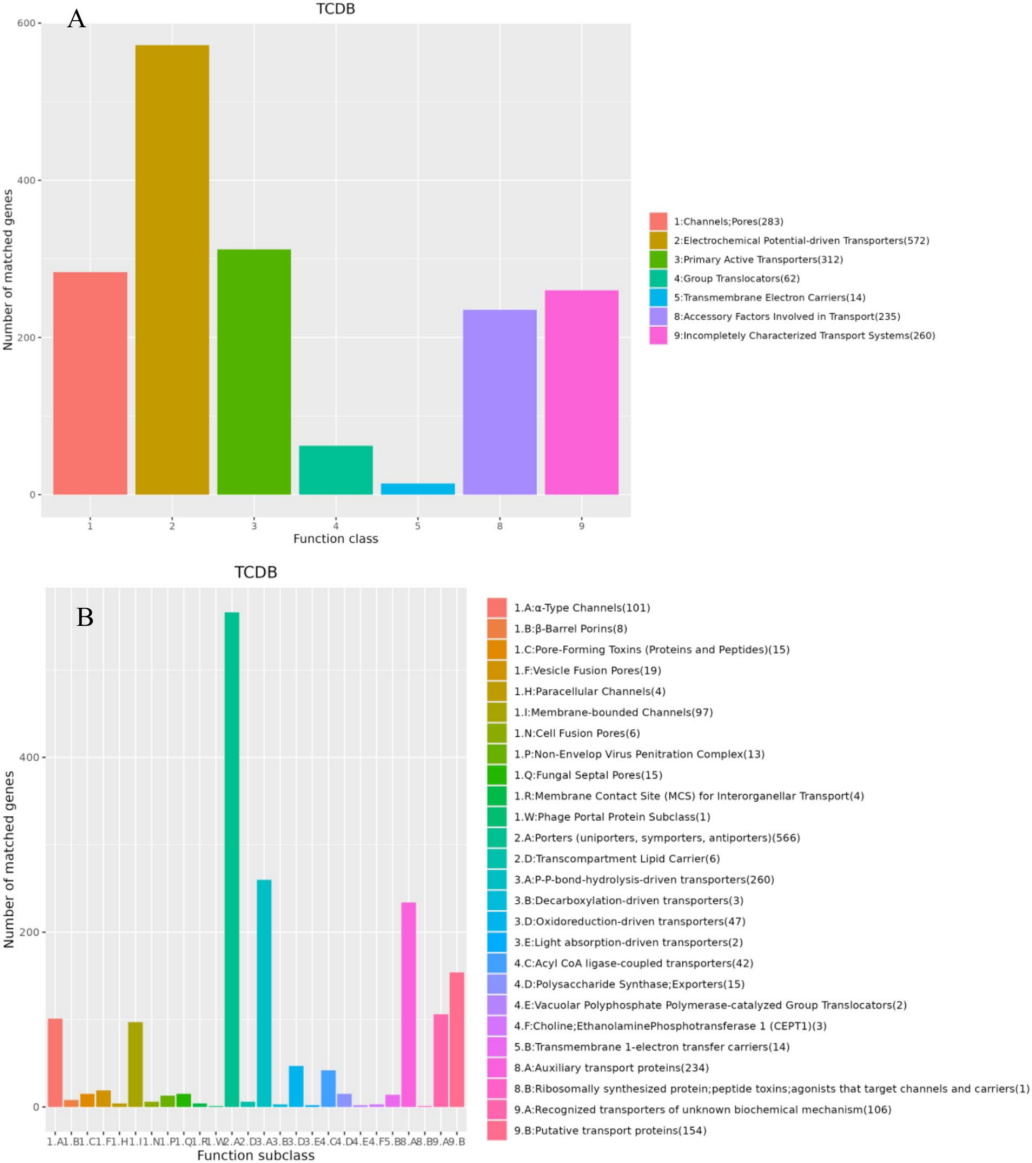
TCDB annotation species statistics chart. **(A)** Primary classification; **(B)** secondary classification.

The TCDB analysis revealed 572 genes in the “Electrochemical Potential-driven Transporters” category in the primary classification (Figure 6A) and 566 genes in the “Porters (uniporters, symporters, antiporters)” category in the secondary classification (Figure 6B). The annotation data indicated that these genes are closely related to the energy supply, self-metabolism, and toxin secretion capabilities of *B. cinerea* HM-Y1.

### 3.4 The effects of pydiflumetofen on *Botrytis cinerea* growth

**Figure 7:**
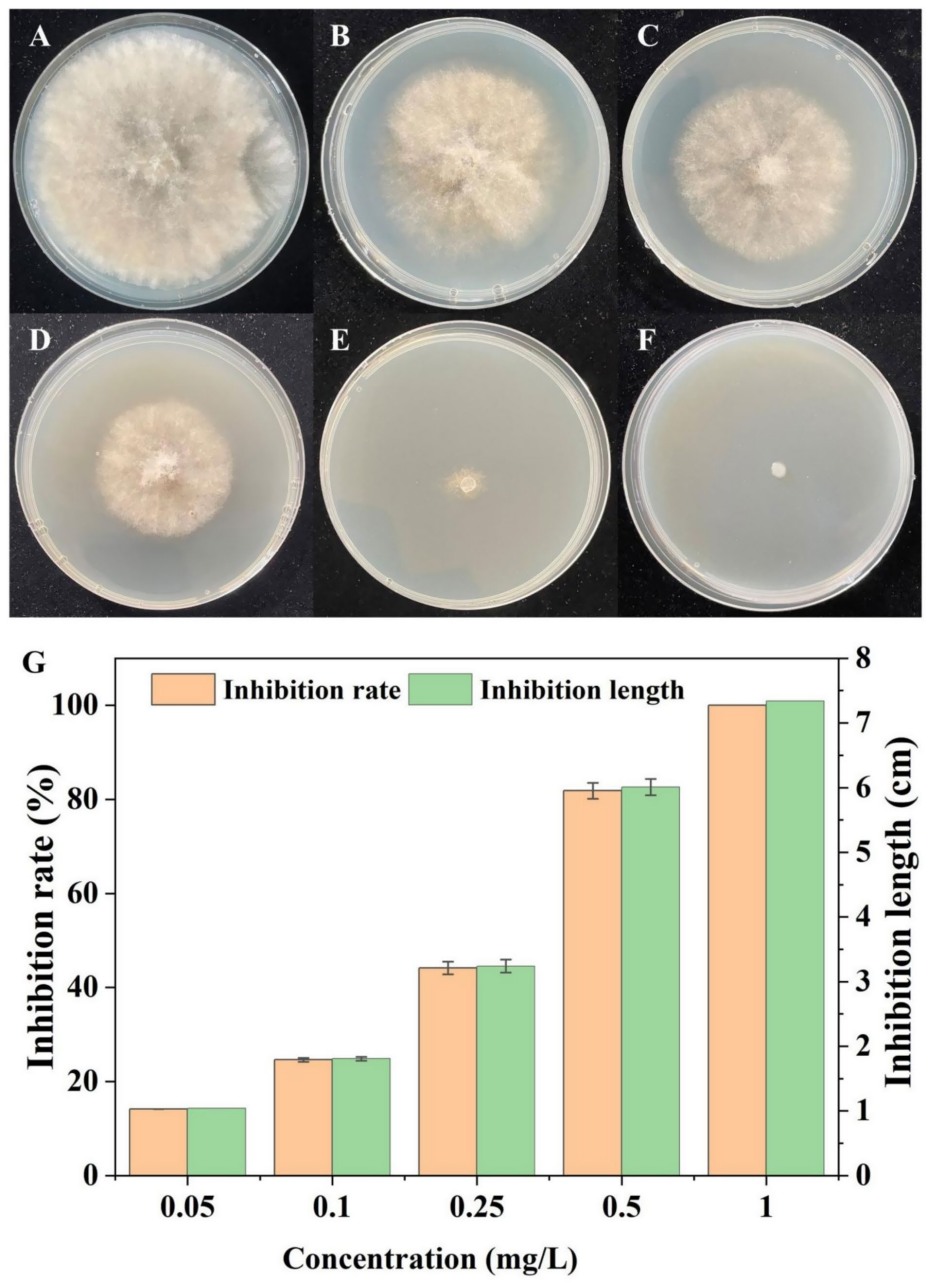
Inhibition effect of pydiflumetofen on *B. cinerea* HM-Y1. Growth status under 0 **(A)**, 0.05 **(B)**, 0.10 **(C)**, 0.25 **(D)**, 0.50 **(E)**, and 1.00 mg/L **(F)** pydiflumetofen; inhibition rate and inhibition length under 0.05, 0.10, 0.25, 0.50, and 1.00 mg/L pydiflumetofen **(G)**.

We next assessed the toxicity of pydiflumetofen on the mycelial growth of *B. cinerea* (Figure 7), and the EC_50_ (concentration) value for the inhibitory effect compared with the control was 0.200 mg/L. The toxicity regression equation for pydiflumetofen was Y = 1.900X + 1.270, with R^2^ = 0.936.

### 3.5 Control effect of pydiflumetofen on *Botrytis cinerea*

**Figure 8:**
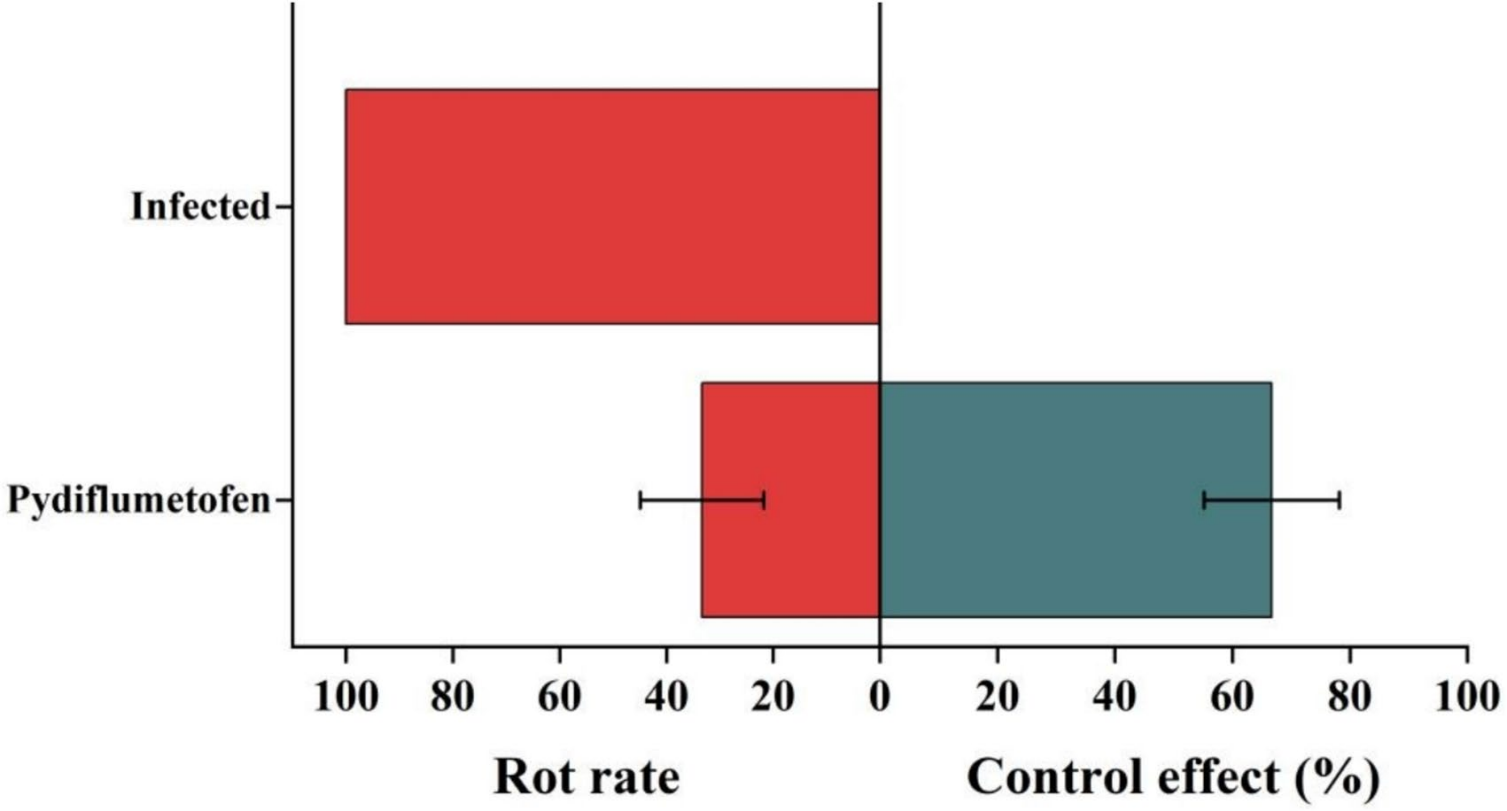
Control effect of pydiflumetofen against postharvest strawberry rot caused by *B. cinerea* HM-Y1.

The control effect of 2 mg/L pydiflumetofen on *B. cinerea* HM-Y1-induced strawberry fruit rot was 66.67% (Figure 8).

## 4 Discussion

Strawberries are susceptible to plant pathogens (Kaur et al., 2025), and storage conditions that increase temperature create favorable conditions for strawberry fruit fungal infections (Lee et al., 2024). High-throughput analysis of the fungal community in strawberry fruits demonstrated that *Botrytis* spp. are the most common pathogens in postharvest rotten strawberries. The sequencing of plant pathogen genomes provides a strong foundation for a deeper understanding of their biological characteristics, pathogenicity, evolutionary mechanisms, and plant interactions. We found multiple sequences of the *ITS, HSP60, RPB2,* and *G3PDH* genes in *B. cinerea* HM-Y1, which indicate *Botrytis* spp. (Li et al., 2023). The gene annotations predicted that these genes are highly pathogenic in this strain. *B. cinerea* can cause rotting in various fruits, such as blueberries (Ghimire et al., 2026), blackberries (Dwivedi et al., 2024), and longans (Guo et al., 2025).

The first genome sequence of *B. cinerea* was published in 2011: a finished genome sequence for the *B. cinerea* strain B05.10 was obtained using a combination of Illumina and PacBio sequencing technologies and optical mapping (Klug et al., 2024). *B. cinerea* secretes a large number of CDIPs and phytotoxic metabolites, several of which have been described as virulence factors (Klug et al., 2024). Phytoalexins damage the cell ultrastructure and disrupt conidial germination in *B. cinerea*. Previous studies have demonstrated that multiple ABC and MFS transporters reduce this damage by removing natural and artificial chemicals via efflux transport mechanisms (Bulasag et al., 2023). Further research into the regulation of these promoter elements may be crucial for a better understanding of *B. cinerea*’s adaptability to a wide range of phytoalexins (Bulasag et al., 2023). Botrydial is necessary for *B. cinerea* plant tissue colonization (El-Shahir et al., 2024; Vignatti et al., 2020) and is a strain-dependent virulence factor; thus, highly aggressive strains retain substantial virulence even when botrydial synthesis is impaired. The phytotoxin botrydial triggers phosphatidic acid (PA) production in tomato cell suspensions by activating phospholipase D (PLD) and phospholipase C and diacylglycerol kinase (PLC/DGK) (D’Ambrosio et al., 2018). PLC/DGK-derived PA is partially required for botrydial-induced ROS generation. PA is a phospholipid second messenger that induces plant defense responses and is generated by either the PLD or PLC/DGK pathway (D’Ambrosio et al., 2018). Furthermore, botrydial is a phytotoxic sesquiterpene produced by the necrotrophic fungus *B. cinerea* that induces diverse plant defense responses, including ROS production (D’Ambrosio et al., 2018). By understanding the existing or potential virulence factors in pathogenic fungus, researchers can better clarify the pathogenic mechanism by which the pathogenic fungus invades the host. Predicted virulence factors of HM-Y1 will be explored by transcriptomic, metabolomic, gene expression, and functional knockout evidence in the next work.

Consequently, it is important to document such events and develop management practices promptly to reduce the risk of significant crop loss (Kaur et al., 2025; Lu et al., 2025). Pydiflumetofen, a novel SDHI fungicide, exhibited strong inhibitory activity against the growth of mycelium and germination of conidia of *B. cinerea*. However, to delay the development of resistance, pydiflumetofen should be used carefully, preferably alternately or as a mixture (Zhao et al., 2022). Overall, the diversity of strategies employed by fungal pathogens to overcome plant defenses highlights the complex and dynamic nature of plant–pathogen interactions. The study is based on samples collected from a single location and a small number of biological replicates (*n* = 5). It limits the representativeness of the microbiome analysis. However, the conclusions regarding pathogen and antifungal assays dominance should be supported with abundant sampling. It is hypothesized that the use of biological control microorganisms will become more effective in controlling strawberry rot in the future (Yan et al., 2021; Yang et al., 2024).

## Data Availability

The datasets presented in this study can be found in online repositories. The names of the repository/repositories and accession number(s) can be found in the article/supplementary material.

## References

[ref1] Bezerra M. Ribeiro M. Cosme F. Nunes F. M. (2024). Overview of the distinctive characteristics of strawberry, raspberry, and blueberry in berries, berry wines, and berry spirits. Compr. Rev. Food Sci. Food Saf. 23:e13354. doi: 10.1111/1541-4337.13354, 38682687

[ref2] Bulasag A. S. Camagna M. Kuroyanagi T. Ashida A. Ito K. Tanaka A. . (2023). *Botrytis cinerea* tolerates phytoalexins produced by Solanaceae and Fabaceae plants through an efflux transporter BcatrB and metabolizing enzymes. Front. Plant Sci. 14:1177060. doi: 10.3389/fpls.2023.1177060, 37332725 PMC10273015

[ref3] Chen Y. Z. Chen Y. Yang J. (2026). Whole-genome sequencing of pathogenic Nigrospora musae ST1 causing leaf spot disease in *Idesia polycarpa*. J. Fungi 12:226. doi: 10.3390/jof12030226 PMC13027451 41893158

[ref4] Chen S. Wu L. Li Y. Zhao Z. Qi Z. (2024). Baseline sensitivity and resistance analysis of *Botrytis cinerea* to pydiflumetofen in Liaoning Province, China. Eur. J. Plant Pathol. 170, 67–77. doi: 10.1007/s10658-024-02880-7

[ref5] Coca-Ruiz V. Suárez I. Aleu J. Cantoral J. M. González C. Garrido C. . (2024). Unravelling the function of the sesquiterpene cyclase STC3 in the lifecycle of *Botrytis cinerea*. Int. J. Mol. Sci. 25:5125. doi: 10.3390/ijms25105125, 38791163 PMC11120764

[ref6] D’Ambrosio J. M. Gonorazky G. Sueldo D. J. Moraga J. Palma A. A. D. Lamattina L. . (2018). The sesquiterpene botrydial from *Botrytis cinerea* induces phosphatidic acid production in tomato cell suspensions. Planta 247, 1001–1009. doi: 10.1007/s00425-018-2843-8, 29340795

[ref7] Duan X. Wang K. Tang R. Liu J. Cheng K. Gao G. . (2025). Recent advances in biosynthesis and regulation of strawberry anthocyanins. Hortic. Res. 12:uhaf135. doi: 10.1093/hr/uhaf135, 40687928 PMC12272852

[ref8] Dwivedi M. Singh P. Pandey A. K. (2024). Botrytis fruit rot management: what have we achieved so far? Food Microbiol. 122:104564. doi: 10.1016/j.fm.2024.104564, 38839226

[ref9] El-Shahir A. A. Alzamel N. M. Abuzaid A. O. Loutfy N. Alwaleed E. A. (2024). Antifungal properties of *Sargassum cinereum* and *Padina boergesenii* extracts against Fungi associated with strawberry fruits concerning mycotoxin production. Plants 13:3115. doi: 10.3390/plants13223115, 39599324 PMC11597142

[ref10] Gaston A. Osorio S. Denoyes B. Rothan C. (2020). Applying the *Solanaceae* strategies to strawberry crop improvement. Trends Plant Sci. 25, 130–140. doi: 10.1016/j.tplants.2019.10.003, 31699520

[ref11] Ghimire L. Wang Y. Adunola P. Boukari W. Casorzo G. Enciso-Rodriguez F. . (2026). Integrative GWAS and transcriptomic analyses reveal markers and candidate genes associated with resistance to *Botrytis cinerea* fruit rot in blueberry. Hortic. Res. uhag092. doi: 10.1093/hr/uhag092 42312043 PMC13271799

[ref12] Guo X. Yang R. Han Q. Jin J. Zhang Y. Xu Y. (2025). First report of postharvest fruit rot in longan caused by *Botrytis cinerea* in China. Plant Dis. 109:2220. doi: 10.1094/pdis-04-25-0796-pdn

[ref13] He L. Cui K. Song Y. Li T. Liu N. Mu W. . (2020). Activity of the novel succinate dehydrogenase inhibitor fungicide Pydiflumetofen against SDHI-sensitive and SDHI-resistant isolates of *Botrytis cinerea* and efficacy against Gray Mold. Plant Dis. 104, 2168–2173. doi: 10.1094/PDIS-12-19-2564-RE, 32526154

[ref14] Hernández-Martínez N. Blanchard C. Wells D. Salazar-Gutiérrez M. R. (2023). Current state and future perspectives of commercial strawberry production: a review. Sci. Hortic. 312:111893. doi: 10.1016/j.scienta.2023.111893

[ref15] Jiang N. Voglmayr H. Xue H. Piao C. G. Li Y. (2022). Morphology and phylogeny of Pestalotiopsis (*Sporocadaceae*, *Amphisphaeriales*) from *Fagaceae* leaves in China. Microbiol. Spectrum 106:e0327222. doi: 10.1128/spectrum.03272-22 PMC9769744 36354327

[ref16] Jiang L. Yan H. Yin Y. Dong R. Wu C. (2025). Use of *Bacillus velezensis* JNS-1 from vermicompost against strawberry root rot. Front. Microbiol. 16:1566949. doi: 10.3389/fmicb.2025.1566949, 40336835 PMC12055544

[ref17] Kaur H. Wesche J. Gelain J. Cai M. Luo C. Schnabel G. (2025). Characterization and fungicide sensitivity of *Gnomoniopsis fructicola* causing Gnomonia leaf blotch of strawberry in the Carolinas. Plant Dis. 109, 107–114. doi: 10.1094/PDIS-07-24-1361-RE, 39172498

[ref18] Kelanne N. M. da Silva C. V. Laaksonen O. Haikonen T. Yang B. Kortesniemi M. (2025). Towards new properties of strawberry: chemical composition and sensory properties of species-reconstructed garden strawberry progenies. Food Chem. 483:144233. doi: 10.1016/j.foodchem.2025.144233 40220444

[ref19] Klug K. Zhu P. Pattar P. Mueller T. Safari N. Sommer F. . (2024). Genome comparisons between *Botrytis fabae* and the closely related gray mold fungus *Botrytis cinerea* reveal possible explanations for their contrasting host ranges. J. Fungi 10:216. doi: 10.3390/jof10030216, 38535224 PMC10971195

[ref20] Lee B. Chen P. Chen C. (2024). Suppression of strawberry anthracnose by *Paenibacillus polymyxa* TP3 in situ and from a distance. Plant Dis. 108, 700–710. doi: 10.1094/PDIS-08-23-1499-RE, 37580883

[ref21] Li X. Gao X. Hu S. Hao X. Li G. Chen Y. . (2022). Resistance to pydiflumetofen in *Botrytis cinerea*: risk assessment and detection of point mutations in sdh genes that confer resistance. Pest Managt Sci. 78, 1448–1456. doi: 10.1002/ps.6762, 34927349

[ref22] Li S. Huang J. W. Min J. Li H. Ning M. Zhou S. . (2025). Molecular insights into a distinct class of terpenoid cyclases. Nat. Commun. 16:207. doi: 10.1038/s41467-024-55717-6, 39747870 PMC11695735

[ref23] Li J. Liu Q. Zhou C. Xie J. (2025). Antifungal activity, physiological disruption, and toxicity mechanisms of difenoconazole in *sclerotinia sclerotiorum*. Crop Prot. 197:107329. doi: 10.1016/j.cropro.2025.107329

[ref24] Li Z. Yu X. Zhang W. Han R. Zhang J. Ma Y. . (2023). Identification, characterization, and pathogenicity of fungi associated with strawberry fruit rot in Shandong Province, China. Plant Dis. 107, 3773–3782. doi: 10.1094/PDIS-04-23-0696-RE, 37408124

[ref25] Li Y. Yue S. Li P. Zeng J. Guo J. Xiong D. . (2025). Genome sequencing and comparative genomics of the hyper-cellulolytic fungus *Talaromyces pinophilus* Y117. J. Fungi 11:614. doi: 10.3390/jof11090614, 41003160 PMC12471171

[ref26] Lin Y. Sun Z. Wang X. Cao S. Liu Y. Liu S. . (2025). Exogenous proanthocyanidins improved postharvest quality and resistance to *Botrytis cinerea* infection of strawberry via enhanced non-enzymatic antioxidants accumulation and salicylic acid signaling pathway. Postharvest Biol. Technol. 230:113732. doi: 10.1016/j.postharvbio.2025.113732

[ref27] Liu Z. Liang T. Kang C. (2023). Molecular bases of strawberry fruit quality traits: advances, challenges, and opportunities. Plant Physiol. 193, 900–914. doi: 10.1093/plphys/kiad376, 37399254

[ref28] Liu L. Zhao K. Liu Z. (2024). Construction and regulation of the abscisic acid biosynthesis pathway in *Yarrowia lipolytica*. J. Agric. Food Chem. 72, 7299–7307. doi: 10.1021/acs.jafc.4c00223, 38504621

[ref29] Lu H. Repp J. Hu M. (2025). Fungicide resistance in *Botrytis* spp. isolates from the northeast and California strawberry fields. Plant Dis. (In press). doi: 10.1094/PDIS-09-25-1828-RE 41408800

[ref30] Minh B. Q. Schmidt H. A. Chernomor O. Schrempf D. Woodhams M. D. von Haeseler A. . (2020). IQ-TREE 2: new models and efficient methods for phylogenetic inference in the genomic era. Mol. Biol. Evol. 37, 1530–1534. doi: 10.1093/molbev/msaa015, 32011700 PMC7182206

[ref31] Momtaz F. Hardy G. Bayliss K. L. (2025). Cold plasma-mediated inhibition of postharvest fungal communities of strawberries. Postharvest Biol. Technol. 230:113834. doi: 10.1016/j.postharvbio.2025.113834

[ref32] Mukherjee E. Gantait S. (2024). Strawberry biotechnology: a review on progress over past 10 years. Sci. Hortic. 338:113618. doi: 10.1016/j.scienta.2024.113618

[ref33] Nguyen L. T. Schmidt H. A. von Haeseler A. Minh B. Q. (2015). IQ-TREE: a fast and effective stochastic algorithm for estimating maximum-likelihood phylogenies. Mol. Biol. Evol. 32, 268–274. doi: 10.1093/molbev/msu300, 25371430 PMC4271533

[ref34] Priyadarshi R. Jayakumar A. de Souza C. K. Rhim J. Kim J. T. (2024). Advances in strawberry postharvest preservation and packaging: a comprehensive review. Compr. Rev. Food Sci. Food Saf. 23:e13417. doi: 10.1111/1541-4337.13417 39072989

[ref35] Qiu C. W. Zhang S. Gao Z. F. Chen Z. H. Zhang C. Ali M. A. . (2026). First Tetraploa genome and multi-omics analysis reveal key plant-microbe-soil interactions for salt tolerance and yield improvement of wheat. Plant Biotechnol. J. (In press). doi: 10.1111/pbi.70663, 41933506 PMC13387892

[ref36] Shi X. Zhang Y. Yang J. Chen Y. (2024). A genomic sequence resource of *Diaporthe mahothocarpus* GZU-Y2 causing leaf spot blight in *Camellia oleifera*. J. Fungi 10:630. doi: 10.3390/jof10090630, 39330390 PMC11433127

[ref37] Staats M. van Baarlen P. van Kan J. A. L. (2005). Molecular phylogeny of the plant pathogenic genus *Botrytis* and the evolution of host specificity. Mol. Biol. Evol. 22:333. doi: 10.1093/molbev/msi020 15496556

[ref38] Vignatti P. Gonzalez M. E. Jofré E. C. Bolívar-Anillo H. J. Moraga J. Viaud M. . (2020). Botrydial confers *Botrytis cinerea* the ability to antagonize soil and phyllospheric bacteria. Fungal Biol. 124, 54–64. doi: 10.1016/j.funbio.2019.11.003, 31892377

[ref39] Wang L. Jiang S. Zhou C. Li D. Sun C. Dai S. (2025). Exploring novel preservation strategies for blue honeysuckle through high-throughput sequencing and bioinformatics analysis. Postharvest Biol. Technol. 219:113251. doi: 10.1016/j.postharvbio.2024.113251

[ref40] Wang Z. Y. Shen A. Q. Ge Y. X. Zhou C. L. Qiao Y. S. Xiong A. S. . (2025). Regulation of fruit quality formation in strawberry: from omics to biotechnology. PeerJ. 13:e19497. doi: 10.7717/peerj.19497, 40458554 PMC12129004

[ref41] Wang Y. Shu D. Li Z. Luo D. Yang J. Chen D. . (2024). Engineering strategies for enhanced 1′, 4′-trans-ABA diol production by *Botrytis cinerea*. Microb. Cell Factories 23:185. doi: 10.1186/s12934-024-02460-8, 38926702 PMC11210036

[ref42] White T. J. Bruns T. Lee S. Taylor J. (1990). “Amplification and direct sequencing of fungal ribosomal RNA genes for phylogenetics,” in PCR Protocols: A Guide to Methods and Applications, vol. 18. eds. Innis M. A. Gelfand D. H. Sninsky J. J. White T. J. (New York, NY: Academic Press Inc.). 315–322. doi: 10.1016/B978-0-12-372180-8.50042-1

[ref43] Xing K. Yang S. Tao J. Liang S. Y. Kong S. Y. Qin S. (2025). Volatile organic compounds of the solid fumigant prepared by strain *Streptomyces setonii* WY228 control gray mold disease in postharvest strawberries. Postharvest Biol. Technol. 229:113715. doi: 10.1016/j.postharvbio.2025.113715

[ref44] Xu H. Quan Q. Xie Y. Xu S. Chang X. Wang R. . (2024). Unraveling and comparing bacterial community signatures and functions of postharvest strawberries packaged with different films during storage. LWT 199:116078. doi: 10.1016/j.lwt.2024.116078

[ref45] Yan H. Jiang L. Guo T. Motelica-Heino M. Wu C. (2026). Emergence of postharvest strawberry fruit rot caused by *penicillium citrinum* in China and its whole-genome sequencing. J. Fungi 12:288. doi: 10.3390/jof12040288, 42042382 PMC13117804

[ref46] Yan H. Li Y. Lv Y. Zhao X. Man Z. Chen Z. . (2025a). Postharvest fruit rot on blue honeysuckle (*Lonicera caerulea* L.) caused by *penicillium oxalicum* newly reported in China. Crop Prot. 193:107195. doi: 10.1016/j.cropro.2025.107195

[ref47] Yan H. Li Y. Man Z. Lv Y. Zhao X. Chen Z. . (2025b). First report of postharvest fruit rot caused by *aspergillus ochraceus* on blue honeysuckle (*Lonicera caerulea* L.) fruit in China. Plant Dis. 109:1173. doi: 10.1094/pdis-12-24-2557-pdn

[ref48] Yan H. Mi Y. Li Y. Zang H. Guo L. Huo J. . (2024). First report of postharvest fruit rot caused by Botrytis cinerea on blue honeysuckle (*Lonicera caerulea*) fruit in China. Plant Dis. 108:527. doi: 10.1094/pdis-08-23-1673-pdn

[ref49] Yan H. Qiu Y. Yang S. Wang Y. Wang K. Jiang L. . (2021). Antagonistic activity of *Bacillus velezensis* SDTB038 against *Phytophthora infestans* in potato. Plant Dis. 105, 1738–1747. doi: 10.1094/PDIS-08-20-1666-RE, 33174798

[ref50] Yang W. Wang M. Wang H. Zhang C. Zhang Q. Xiao H. (2024). Exploitation of the biocontrol potential of a marine-derived bacillus velezensis and its application on postharvest strawberry. Food Control 161:110311. doi: 10.1016/j.foodcont.2024.110311

[ref001] Yousef S. A. M. Ali A. M. Elsherbiny E. A. Atwa A. A. (2024). Morphological, genetic and pathogenic variability among Botrytis cinerea species complex causing gray mold of strawberry. Physiol Mol Plant P 134:102395.

[ref51] Yuan S. Wang B. Wang M. Sun M. Wang X. Li X. . (2024). Antifungal mechanism of protocatechuic acid methyl ester against Botrytis cinerea in postharvest strawberry fruit. Postharvest Biol. Technol. 211:112787. doi: 10.1016/j.postharvbio.2024.112787

[ref52] Yuan H. Wang L. Zhu J. Ren J. Liu R. Li X. . (2025). Inhibitory effect of 2-methylvaleraldehyde on *Botrytis cinerea* and its application in postharvest strawberry. Postharvest Biol. Technol. 230:113775. doi: 10.1016/j.postharvbio.2025.113775

[ref53] Zhao J. Wu J. Lu F. Bi Q. Yang K. Han X. . (2022). Baseline-sensitivity of *Botrytis cinerea* to pydiflumetofen and its efficacy on tomato gray mold in Hebei Province, China. Crop Prot. 158:105989. doi: 10.1016/j.cropro.2022.105989

[ref54] Zhao L. Xiao Y. Tong H. Jiang S. Zhou Y. Dhanasekaran S. . (2026). Biological control of postharvest soft rot of strawberries by *Wickerhamomyces anomalus* and the involved mechanisms. Food Microbiol. 134:104927. doi: 10.1016/j.fm.2025.104927, 41136144

[ref55] Zhao L. Zhou Y. Liang L. Godana E. A. Zhang X. Yang X. . (2023). Changes in quality and microbiome composition of strawberry fruits following postharvest application of *Debaryomyces hansenii*, a yeast biocontrol agent. Postharvest Biol. Technol. 202:112379. doi: 10.1016/j.postharvbio.2023.112379

[ref56] Zhou L. Liu Y. Kong F. Jia S. Wang Q. Wang Z. . (2024). Sensitivity of *Botrytis cinerea* from vineyards to boscalid, isofetamid, and pydiflumetofen in Shandong Province, China. Phytopathology 114, 1068–1074. doi: 10.1094/PHYTO-10-23-0369-KC, 38105240

